# Association of Bleeding Scores and Platelet Transfusions With Platelet Counts and Closure Times in Response to Adenosine Diphosphate (CT-ADPs) Among Preterm Neonates With Thrombocytopenia

**DOI:** 10.1001/jamanetworkopen.2020.3394

**Published:** 2020-04-27

**Authors:** Emoke Deschmann, Matthew A. Saxonhouse, Henry A. Feldman, Mikael Norman, Maria Barbian, Martha Sola-Visner

**Affiliations:** 1 Division of Neonatology, Department of Women’s and Children’s Health, Karolinska Institutet, Stockholm, Sweden; 2 Department of Neonatology, Karolinska University Hospital, Stockholm, Sweden; 3 Division of Newborn Medicine, Boston Children’s Hospital, Harvard Medical School, Boston, Massachusetts; 4 Division of Neonatology, Levine Children’s Hospital, Atrium Healthcare, University of North Carolina School of Medicine, Charlotte; 5 Institutional Centers for Clinical and Translational Research, Boston Children’s Hospital, Boston, Massachusetts; 6 Division of Pediatrics, Department of Clinical Science, Intervention, and Technology, Karolinska Institutet, Stockholm, Sweden

## Abstract

This cohort study analyzes the association of closure time in response to adenosine diphosphate (CT-ADP) with bleeding score and the associations of platelet transfusions with change in platelet count, CT-ADP, and bleeding scores in preterm neonates with thrombocytopenia.

## Introduction

Platelet transfusions (PTX) are frequently given to neonates with thrombocytopenia to prevent bleeding.^1,2^ However, there is a poor association between platelet counts (PCs) and bleeding in neonates,^2,3,4^ suggesting that other factors are more important for bleeding risk than PCs.

We previously showed^5^ that closure times in response to adenosine diphosphate (CT-ADPs), an in vitro test of primary hemostasis, were associated with concurrent bleeding scores (BSs) in preterm neonates, while PCs were not.^5^ In the current study, we analyzed the association of CT-ADP with BS over time and the associations of PTX with changes in PC, CT-ADP, and BS in neonates younger than 27 weeks’ gestational age.

## Methods

The Neonatal Hemorrhagic Risk Assessment in Thrombocytopenia Study was a prospective longitudinal study conducted in 2 neonatal intensive care units (Karolinska University Hospital and Levine Children’s Hospital). The study was approved by the Central Ethical Review Board in Sweden and by the Atrium Healthcare institutional review board. Written informed consent was obtained from parents. This study followed Strengthening the Reporting of Observational Studies in Epidemiology (STROBE) reporting guideline.

A total of 76 neonates (mean [SD] gestational age, 26.1 [2.4] weeks; mean [SD] birth weight, 777 [310] g; median [interquartile range] postnatal age, 4 [3-14] days) were enrolled in the Neonatal Hemorrhagic Risk Assessment in Thrombocytopenia Study. Patients were eligible if they were less than 32 weeks’ gestation or weighed less than 1500 g at birth, had a PC less than 100 × 10^3^/µL (to convert to ×10^9^/L, multiply by 1.0), and had written informed consent from a parent or guardian. Patients were excluded if they were not expected to survive, were thought to have a congenital thrombocytopenia or platelet dysfunction, or had a major chromosomal anomaly. Eligible neonates were enrolled between May 2015 and September 2017,^5^ and PC, CT-ADP, and BS were measured on 3 consecutive days in each infant. Bleeding was quantified with the Neonatal Bleeding Assessment Tool, providing a BS ranging from 0 (no bleeding) to 4 (major bleeding). Details of all methods have been published previously.^5^

Ordinal multinomial logistic regression was used to quantify the association of BS (grouped 0-1, 2, and 3-4) with the prior day’s CT-ADP. To assess the association of 1-day changes in BS, CT-ADP, and PC with incident transfusion, we used linear regression and correlation. All analyses were conducted in SAS version 9.4 (SAS Institute), and we used repeated-measures models to account for within-patient covariance. Statistical significance was set at *P* < .05, and all tests were 2-tailed.

## Results

Baseline characteristics of the Neonatal Hemorrhagic Risk Assessment in Thrombocytopenia cohort have been reported previously.^5^ Among the 54 patients with gestational age less than 27 weeks in the current study (mean [SD] gestational age, 24.8, [1.1] weeks; mean [SD] birth weight, 662 [151] g), a longer CT-ADP was associated with a higher probability of grade 2 to grade 4 bleeding the following day, rising from 10% after minimum CT-ADP (ie, 0 seconds) to 40% after maximum CT-ADP (ie, 300 seconds). Each 60-second increment in CT-ADP was associated with greater odds of higher BS the next day (odds ratio, 1.42; 95% CI, 1.01-2.00; *P* = .04), but adjustment for PC reduced the estimate of effect size (odds ratio, 1.33; 95% CI, 0.96-1.87; *P* = .09).

Changes in CT-ADP were strongly correlated with changes in BS (*r* = 0.33; *P* = .008), while changes in PC were not (*r* = –0.01; *P* = .93) (Figure). In regression analyses, changes in CT-ADP were associated with BS changes, with a 0.24-point (95% CI, 0.12-0.37 points) increase in BS per 60-second increase in CT-ADP (*P* < .001). This association was unaffected by adjustment for PC changes (0.26-point [95% CI, 0.12-0.40 points] increase in BS per 60-second increase in CT-ADP; *P* < .001) or transfusions (0.25-point [95% CI, 0.12-0.37 points] increase in BS per 60-second increase in CT-ADP; *P* < .001).

**Figure.**
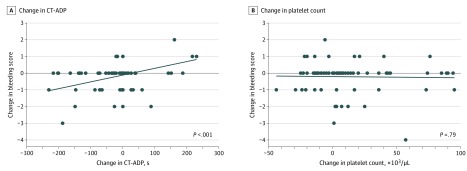
Association of Changes in Bleeding Scores and Changes in Closure Time in Response to Adenosine Diphosphate (CT-ADP) or Platelet Count Graphs show 1-day changes in bleeding scores were associated with changes in CT-ADP (*P* < .001) but not with changes in platelet count (*P* = .79) in infants with gestational age of less than 27 weeks. Solid line indicates fitted regression line, and dots represent individual measurements. NeoBAT indicates Neonatal Bleeding Assessment Tool. To convert platelet count to ×10^9^/L, multiply by 1.0.

A total of 17 patients (31.5%) received 21 PTX at a mean (SD) PC of 49 (21) × 10^3^/μL. They exhibited significant 1-day increases in PC (mean [SD] change, 31 [9]; *P* = .002) and decreases in CT-ADP (mean [SE] change, −51 [22]; *P* = .03) but no significant changes in BS. Fresh frozen plasma (9 transfusions given to 8 infants [14.8%]) or erythrocyte transfusions were not associated with CT-ADP or BS changes (Table).

**Table.** One-Day Change in PC, CT-ADP, and NeoBAT Score by Type of Transfusion in Infants With Gestational Age Less Than 27 Weeks

Outcome	Infants receiving transfusion^a^			Infants not receiving transfusion^a^			*P* value^b^
	Sample pairs	Mean (SE)	*P* value	Sample pairs	Mean (SE)	*P* value	
**Platelet transfusion**							
Change in PC, ×10^3^/μL	21	31 (9)	.002	55	11 (6)	.06	.06
Change in CT-ADP, s	20	–51 (22)	.03	47	–11 (14)	.44	.14
Change in bleeding score							
All infants	29	–0.28 (0.18)	.12	79	–0.11 (0.11)	.29	.44
Infants with ≥2 bleeding score on first day	13	–0.54 (0.32)	.12	23	–0.65 (0.24)	.02	.78
**Fresh frozen plasma transfusion**							
Change in CT-ADP, s	9	–10 (33)	.76	58	–25 (13)	.06	.68
Change in bleeding score							
All infants	11	–0.27 (0.29)	.35	97	–0.14 (0.10)	.14	.67
Infants with ≥2 bleeding score on first day	6	–0.17 (0.47)	.73	30	–0.70 (0.21)	.005	.32
**Erythrocyte transfusion**							
Change in hematocrit, %	28	–0.6 (1.0)	.56	44	–1.3 (0.8)	.14	.63
Change in CT-ADP, s	27	–23 (19)	.23	40	–23 (15)	.16	.98
Change in bleeding score							
All infants	31	–0.26 (0.17)	.14	77	–0.12 (0.11)	.29	.49
Infants with ≥2 bleeding score on first day	9	–1.00 (0.38)	.02	27	–0.48 (0.22)	.05	.26

Abbreviations: CT-ADP, closure time in response to adenosine diphosphate; NeoBAT, Neonatal Bleeding Assessment Tool; PC, platelet count.

SI conversion factors: To convert hematocrit to proportion of 1.0, multiply by 0.01; PC to ×10^9^/L, multiply by 1.0.

^a^ Mean (SE) 1-day change was adjusted for within-patient correlation. *P* tests for mean change of 0.

^b^ Comparing mean change on days with or without transfusions.

## Discussion

The main finding of this study was an association between changes in BS and changes in CT-ADP (but not PC), suggesting that primary hemostasis and bleeding are dynamic and more interconnected than PC and bleeding in extremely preterm neonates with thrombocytopenia. Limitations of our study included the small number of infants with PCs less than 50 × 10^3^/µL because of platelet transfusion practices in our neonatal intensive care units and the relatively small number of the more clinically relevant bleeding grades (ie, 3-4). The limited number of CT-ADPs obtained in each infant was because of the relatively high blood volume required by the test (800 µL). We also recognize that all associations presented may be subject to residual or unmeasured confounding variables.

Platelet transfusions given at the thresholds used in this study increased PCs but did not reduce BSs. This was consistent with the Platelet Transfusion Thresholds in Premature Neonates (PlaNeT-2) study, in which PTX for PCs less 50 × 10^3^/μL did not reduce bleeding.^6^ Together, these findings suggest that when PCs are not extremely low, other factors could be contributors to bleeding in neonates with thrombocytopenia, although the PC threshold below which a PTX reduces bleeding is unknown. Overall, 8 infants in our cohort also received 9 fresh frozen plasma transfusions for clinical bleeding. However, these transfusions did not reduce CT-ADP or BS and did not affect their association. Implementing CT-ADP or other measures of primary hemostasis in clinical practice may lead to novel approaches to manage thrombocytopenia in preterm neonates.
